# A post-weaning obesogenic diet exacerbates the detrimental effects of maternal obesity on offspring insulin signaling in adipose tissue

**DOI:** 10.1038/srep44949

**Published:** 2017-03-24

**Authors:** Juliana de Almeida Faria, Daniella Duque-Guimarães, Asha A. M. Carpenter, Elena Loche, Susan E. Ozanne

**Affiliations:** 1 University of Cambridge Metabolic Research Laboratories and MRC Metabolic Diseases Unit, Wellcome Trust-MRC Institute of Metabolic Science, Addenbrooke’s Hospital, Cambridge, CB2 0QQ, United Kingdom; 2 University of Campinas, Faculty of Medical Sciences, Department of Pharmacology, Campinas, 13083-894, Brazil; 3 University of São Paulo, Institute of Biomedical Sciences, Department of Physiology and Biophysics, São Paulo, 05508-000, Brazil

## Abstract

Previous studies have shown that maternal diet-induced obesity leads to increased risk of type 2 diabetes in offspring. The current study investigated if weaning onto an obesogenic diet exaggerated the detrimental effects of maternal diet-induced obesity in adipose tissue. Maternal obesity and offspring obesity led to reduced expression of key insulin signalling proteins, including insulin receptor substrate-1 (IRS-1). The effects of maternal obesity and offspring obesity were, generally, independent and additive. Irs1 mRNA levels were similar between all four groups of offspring, suggesting that in both cases post-transcriptional regulation was involved. Maternal diet-induced obesity increased miR-126 expression however levels of this miR were not influenced by a post-weaning obesogenic diet. In contrast, a post-weaning obesogenic diet was associated with increased levels of suppressor of cytokine signaling-1, implicating increased degradation of IRS-1 as an underlying mechanism. Our results suggest that whilst programmed reductions in IRS-1 are associated with increased levels of miR-126 and consequently reduced translation of Irs1 mRNA, the effects of a post-weaning obesogenic diet on IRS-1 are mediated by miR-126 independent mechanisms, including increased IRS-1 protein degradation. These divergent mechanisms explain why the combination of maternal obesity and offspring obesity leads to the most pronounced effects on offspring metabolism.

The obese population has reached epidemic proportions around the world, especially in urban settings. The prevalence of obesity has increased in both genders and in all age groups within the population. However in 2014 the number of obese women, including those of childbearing age, exceeded that of men in all World Health Organization (WHO) regions^1^. Obesity in women during pregnancy is a major concern as studies in rodents and humans have suggested that *in utero* and early postnatal exposure to obesity can have long term “programming” effects^2^.

Obesity is associated with several metabolic disruptions, such as insulin resistance and type-2 diabetes^3^,^4^. As well as current obesity having a major impact on our metabolic health, there is growing evidence that obesity during pregnancy, not only has immediate negative consequences for the mother and her new born infant, but also causes long term detrimental health consequences for the child, including increased risk of type-2 diabetes^5^. In humans, some of the strongest evidence for the importance of the intrauterine environment has come from the study of siblings from pregnancies that occurred pre and post-bariatric surgery in the mother. The authors found that children born post maternal bariatric surgery (and subsequent weight loss) were leaner, more insulin sensitive and had lower blood pressure compared to their siblings born prior to maternal surgery^6^. Similar effects of maternal obesity have been observed in animal studies, showing that mothers fed a junk food diet during pregnancy and lactation had offspring that displayed enhanced preference for salty, sugary and fatty foods^7^. These programming effects are thought to occur through non-genetic (“epigenetic”) mechanisms.

Epigenetics is defined as the environmental influence on gene expression that can alter the phenotype of the cell independently of gene sequence modifications^8^. Among the main epigenetic mechanisms/factors are DNA methylation, histone modifications and small non-coding RNA molecules (such as miRNAs) that can all work separately or in combination to influence gene expression^9^. miRNAs have an essential role in the control of a myriad of biological functions^10^ including lipid/glucose homeostasis^11^,^12^ and mediate their effects through post-transcriptional regulation of gene expression^13^.

We have shown previously that reduction in adipose tissue of insulin signaling protein expression is one mechanism by which maternal diet-induced obesity leads to increased risk of type 2 diabetes mellitus (T2DM) in the offspring^14^. Mouse offspring of dams fed a high fat-high sugar diet during pregnancy developed insulin resistance by 8 weeks of age when weaned onto a low fat chow diet and were lean. This was associated with reduced protein levels of adipose tissue IRS-1 and a parallel increase in miR-126 expression, a miRNA that directly regulates translation of *Irs1* mRNA through binding to its 3′ un-translated region. Importantly these programmed effects on IRS-1 and miR-126 were cell autonomous and were retained in pre-adipocytes from programmed animals that were differentiated *in vitro*^14^.

It is well established that consumption of a high-fat/simple sugar diet *per se* contributes to insulin resistance and pathogenesis of type 2 diabetes^15^,^16^. In most situations, individuals who have been exposed to an obesogenic diet during fetal life will also be exposed to an obesogenic environment during postnatal life. The aim of this study was therefore to determine if weaning offspring onto an obesogenic diet exaggerated the effects of maternal diet-induced obesity on adipose tissue insulin signalling protein expression and if so if obesity during different time windows mediates its effects through different molecular pathways.

## Methods

### Animals and diet

**Figure 1:**
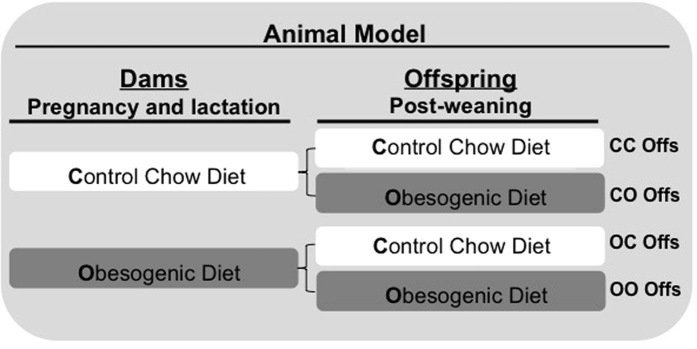
Representative scheme of the animal model used in this study. The offspring were named according to maternal and post-weaning diet. The first letter represents maternal diet (Chow standard or obesogenic) during pregnancy and lactation periods. The second letter represents the post-weaning diet. All results presented are related to metabolic profile found in eight weeks-old offspring.

This study was approved by the University of Cambridge Animal Welfare and Ethical Review Board and was conducted according to the Home Office Animals (Scientific Procedures) Act 1986. It capitalised on an established mouse model of maternal diet-induced obesity^17^, where female C57BL/6 J mice were fed *ad libitum* either a standard chow diet (RM1) (~7% simple sugars, 3% fat, 50% polysaccharide, 15% protein [wt/wt]), or a semi-synthetic energy-rich highly palatable obesogenic diet (~10% simple sugars, 20% animal lard, 28% polysaccharide, 23% protein [wt/wt]) supplemented with sweetened condensed milk (~55% simple sugar, 8% fat, 8% protein [wt/wt], Nestle (Nestle, UK) and fortified with mineral and vitamin mix AIN93G for six weeks before mating. Diets were purchased from Special Dietary Services, Witham, UK. To ensure that dams were proven breeders all dams went through a first pregnancy before re-mating for a second pregnancy. The first day of pregnancy was considered by the appearance of a vaginal plug. Dams received their respective experimental diets during pregnancy and lactation and were weighed at the beginning and end of pregnancy. Litter sizes were culled to 6 on postnatal day 3 by random selection to ensure standardized milk supply. Litters were not disturbed until day 3 as from experience C57BL/6J mothers become stressed and can reject their litters if handled in the first 48 hours after birth. At 21 days of age offspring were weaned onto either the control or obesogenic diet, fed *ad libitum*. This generated four offspring experimental groups: 1 (CC) - Dams and offspring were fed standard chow diet; 2 (OC) - Obesogenic diet was given to dams during pregnancy and lactation and offspring were weaned onto the control standard chow diet; 3 (CO) - Dams were fed the standard chow diet during pregnancy and lactation and offspring were weaned onto the obesogenic diet; and 4 (OO) - Both dams and offspring, were fed the obesogenic diet. (See Fig. 1 for schematic diagram). Body weight was recorded weekly. At eight weeks of age, following a 4 hour fast, blood glucose was measured (OneTouch Ultra; LifeScan Inc., Milpitas, CA) and offspring were killed by raising CO_2_ concentration. The time point of eight weeks of age was chosen based on a previous study from our group, which demonstrated that offspring at this age were insulin resistant as consequence of maternal diet-induced obesity and it happened prior to the development of obesity (14). Epididymal fat was dissected, weighed, snap frozen and stored at −80 °C or fixed in formalin for further analysis. To avoid confounding effects of sex differences in susceptibility to obesity and insulin resistance and to avoid variability caused by hormonal cycles in females, only male mice were included in this study.

### Adipocyte size

Epididymal adipose tissue was fixed in formalin, processed (VIP6, Sakula, USA), embedded in paraffin, sectioned in 5 μm sections and stained using hematoxylin-eosin. One slide was prepared per animal and a 10X magnification was used to quantify the diameter of adipocyte. For each slide, five randomly chosen fields of view from different areas were taken and the adipocyte size obtained was calculated using AxioVision software version 4.8 (Zeiss, Germany).

### Lipid content

Lipid content of epididymal fat was determined using the Folch Assay^18^. Briefly, epididymal fat samples were homogenised in a 2:1 ratio of chloroform:methanol. The distinct lipid phase was removed after centrifugation and lipid weight was quantified after the solvent was removed by overnight evaporation.

### microRNA expression

Total RNA was extracted from epididymal fat samples as previously described^14^ using the Direct-zol™ RNA MiniPrep Plus kit (Zymo Research, USA). For miRNAs miR-27b, miR-29a, miR-126, miR-128 and miR-145, the kits hsa-miR-27b Assay ID: 000409, hsa-miR-29a Assay ID: 002112, hsa-miR-126 Assay ID: 002228, hsa-miR-128a Assay ID: 002216 and hsa-miR-145 Assay ID: 002278 were used, respectively (Applied Biosystems, UK). Standard curves were generated using serial dilution of pooled cDNA samples and microRNA expression was normalized to U6 snRNA (Assay ID: 001973, Applied Biosystems, UK), which displayed no differences between the groups.

### Gene expression

Quantitative RT-PCR was used to determine the relative expression levels of *Irs1* mRNA [as described in ref. 14]. Glyceraldehyde 3-phosphate dehydrogenase (GAPDH) was used as the housekeeping gene as its expression was stable among groups. Expression changes were measured according to the comparative cycle threshold method. Total RNA concentration and purity were evaluated spectrophotometrically using the NanoDrop ND-1000 (Thermo Fisher Scientific). Samples were analysed in duplicate.

### Western blotting

This procedure was performed as described previously^14^. Each membrane was incubated overnight with the respective primary antibody [IRS-1 and Phosphatidylinositol-4,5-bisphosphate 3-kinase p85 alpha subunit (PI3Kp85α) (Upstate Biotechnology, Millipore, Lake Placid, USA), Phosphatidylinositol-4,5-bisphosphate 3-kinase p110 beta subunit (PI3Kp110β), Protein kinase B1 (AKT1), Protein kinase B2 (AKT2), Suppressor of cytokine signaling protein 1 (SOCS1) and Suppressor of cytokine signaling protein 3 (SOCS3) (Cell Signaling, UK) and Insulin receptor beta (IRβ), protein kinase C-ζ (PKCζ) and Glucose transporter type 4 (GLUT4) receptor (Santa Cruz Biotechnology, Germany)]. Following washing with TBS/T solution (1 X TBS, 0.05% Tween 20), membranes were incubated with horseradish peroxidase-conjugated anti-rabbit or anti-mouse antibody as appropriate (Jackson Immuno Research, UK). Antibody binding was detected using Super Signal West Pico Chemiluminescent substrate (Thermo Scientific, UK) with an ImageQuant^TM^ LAS 4000 machine and quantified using ImageQuant^TM^ LAS 4000 software (GE Healthcare, UK). Coomassie Blue staining was used to confirm equal loading and GAPDH (Santa Cruz Biotechnology, Germany) antibody blotted on each membrane to further confirm equal loading.

### Statistical analysis

Data were analysed from one male per litter hence ‘*n*’ refers to the number of litters per group. All data were analysed using a 2-way ANOVA with maternal diet and offspring diet (post weaning diet) as the independent variables. Bonferroni post-hoc analysis was used for comparisons and where P values are reported, an α level <0.05 was considered statistically significant. All data analysis was conducted using Prism 6 (Graphpad, La Jolla, USA).

## Results

### Anthropometric parameters and glycemia of the offspring

**Figure 2:**
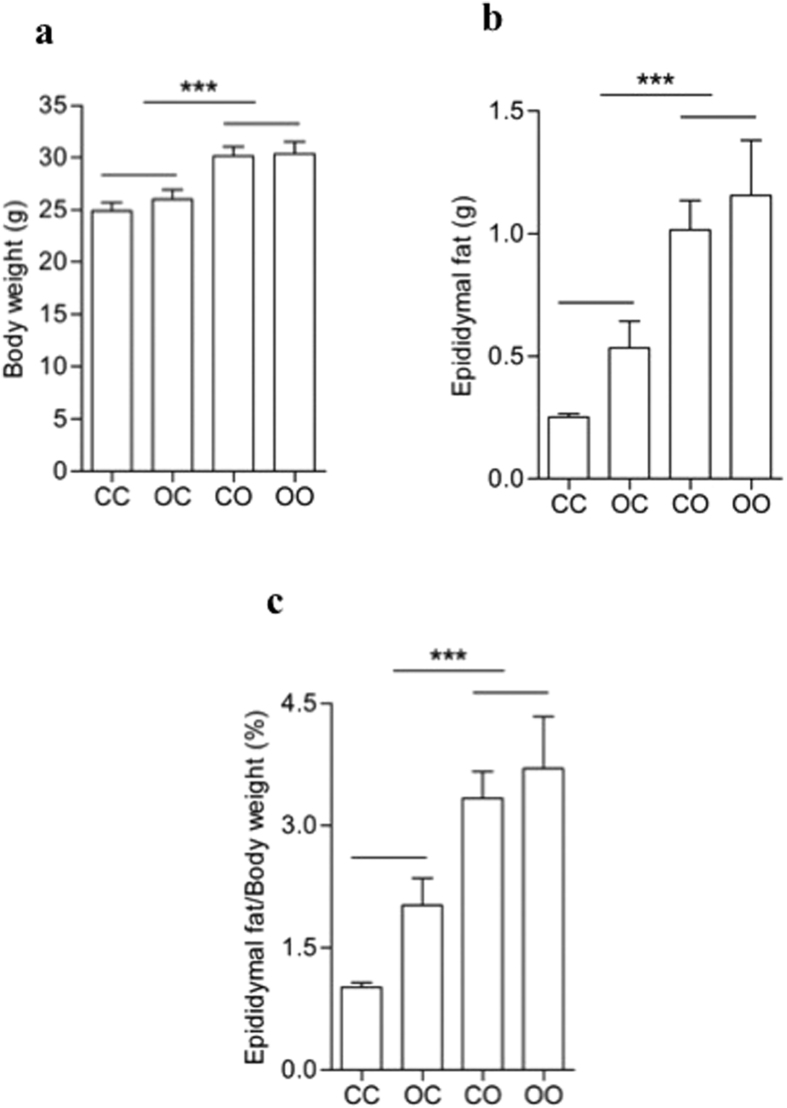
Effects of maternal programming, post-weaning diet and their association on anthropometric parameters of offspring. (**a**) Body weight, (**b**) absolute and (**c**) relative epididymal fat mass which was normalised to total body mass. Bars represent the mean ± SEM (*n* = 6). Effect of offspring diet ***p < 0.001.

At 8 weeks of age, offspring diet but not maternal diet affected the anthropometric parameters of the offspring. As previously described^14^, offspring exposed to maternal obesogenic diet had a similar body weight compared to control offspring (Fig. 2a). However offspring fed a post-weaning obesogenic diet were heavier than those animals fed a control diet post-weaning (Effect of offspring diet: p < 0.001, Fig. 2a). Epididymal white adipose tissue (eWAT) was higher in offspring exposed to a post weaning obesogenic diet compared with controls but there was no effect of maternal diet (Effect of offspring diet: p < 0.001, Fig. 2b). When the eWAT was adjusted for total body weight (Fig. 2c), the pattern was identical to the one described for eWAT absolute values (Effect of offspring diet: p < 0.001). All effects shown were independent, with no significant interaction. There were no differences in fasting glucose levels between any of the four groups (CC = 11.8 ± 0.5, OC = 11.5 ± 0.9, CO = 11.8 ± 0.9 and OO = 12.2 ± 1.1 mmol/L).

### Size of adipocytes and total fat content in the offspring epididymal fat

**Figure 3:**
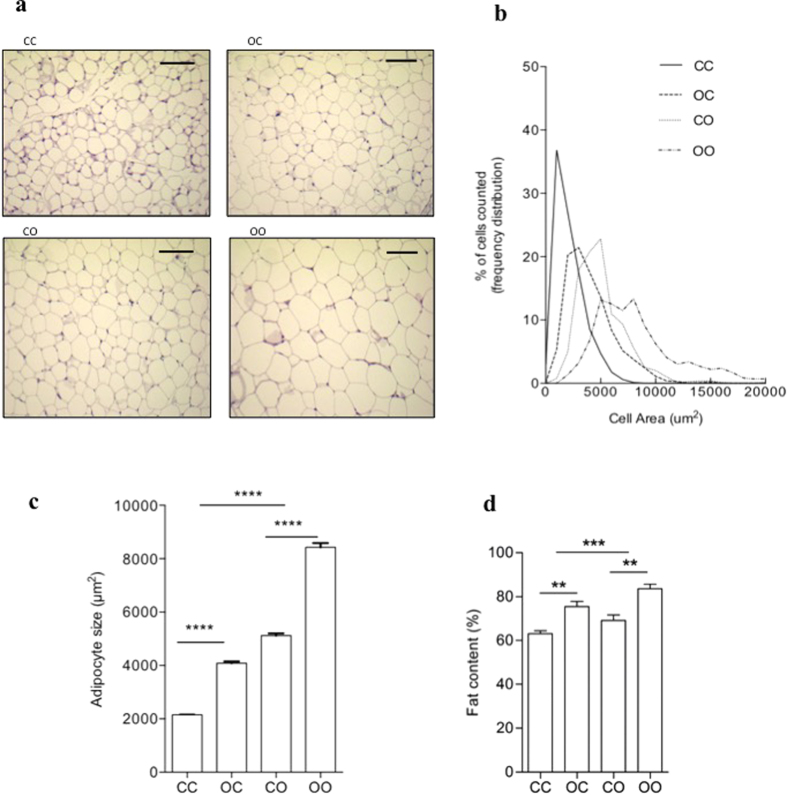
Maternal programming followed by post-weaning obesogenic diet enhance the size of adipocytes and its lipid accumulation. Representative histological images for. (**a**) CC, OC, CO and OO offspring. Scale bar represents 100 μm. (**b**) Frequency distribution of cell groups and (**c**) Diameter of adipocytes (*n* = 5) shows effect of maternal diet ****p < 0.0001 and offspring diet ****p < 0.0001, with significant interaction ****p < 0.0001. (**d**) Total fat content (*n* = 5) shows effect of maternal diet **p < 0.01 and offspring diet ***p < 0.001, with no interaction.

Maternal diet (p < 0.0001) and the post-weaning obesogenic diet (p < 0.0001) both resulted in an increase in eWAT cell size (Fig. 3a – c). This meant that the animals that were exposed to maternal obesity and then consumed an obesogenic diet from weaning had the largest adipocytes (Fig. 3c). In addition, there was interaction (p < 0.0001) between maternal and post-weaning obesogenic diet to generate the enlargement of adipocytes (Fig. 3a – c). Total fat content was increased by maternal (p < 0.01) and offspring diet (p < 0.001) and the effects were additive so the animals that were exposed to maternal obesity and then consumed an obesogenic diet had the greatest lipid content (Fig. 3d).

### Effects of maternal diet-induced obesity and a post-weaning obesogenic diet on adipose tissue insulin signaling protein expression

**Figure 4:**
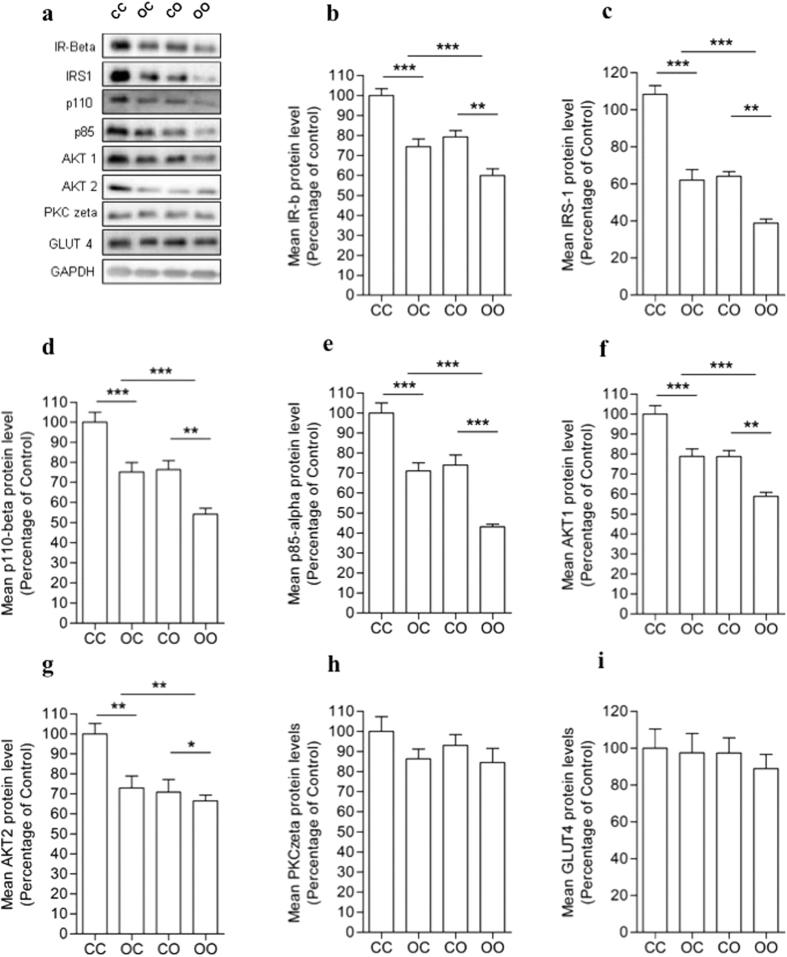
Nutritional programming exacerbates the effects of post-weaning obesogenic diet on insulin signaling pathway proteins. (**a**) Representative image of protein expression. (**b**) IR-beta, (**c**) IRS-1, (**d**) PI3Kp110-beta, (**e**) PI3Kp85, (**f**) AKT1, (**g**) AKT2, (**h**) PKC zeta and (**i**) GLUT4 proteins in eWAT of 8 weeks-old male offspring. IR-beta, IRS-1, PI3Kp110, PI3Kp85 and AKT1 show effect of maternal diet ***p < 0.001, **p < 0.01 and offspring diet ***p < 0.001. AKT2 shows effect of maternal diet **p < 0.01, *p < 0.05 and offspring diet **p < 0.01. All effects shown were independent, with no significant interaction. Data presented as percentage of CC group ± SEM (*n* = 6). Full-length blots are presented in Supplementary Figure 1a.

As shown in the representative image (Fig. 4a) both a maternal (p < 0.001) and post-weaning (p < 0.001) obesogenic diet reduced expression of the following insulin signaling proteins: the insulin receptor (Fig. 4b), IRS-1 (Fig. 4c), p110-beta catalytic subunit of PI 3-knase (Fig. 4d), p85-alpha regulatory subunit of PI 3-kinase (Fig. 4e) and AKT1 (Fig. 4f). The maternal obesogenic diet (p < 0.01) and the offspring obesogenic diet (p < 0.01) also led to a reduction in AKT2 (Fig. 4g). PKC zeta (Fig. 4h) and GLUT4 (Fig. 4i) proteins were not modulated by either a maternal or a post-weaning obesogenic diet, suggesting the effects of maternal obesity and offspring obesity are specific to a subset of insulin signalling proteins.

### The involvement of miRNA-126 in insulin signaling pathway regulation

**Figure 5:**
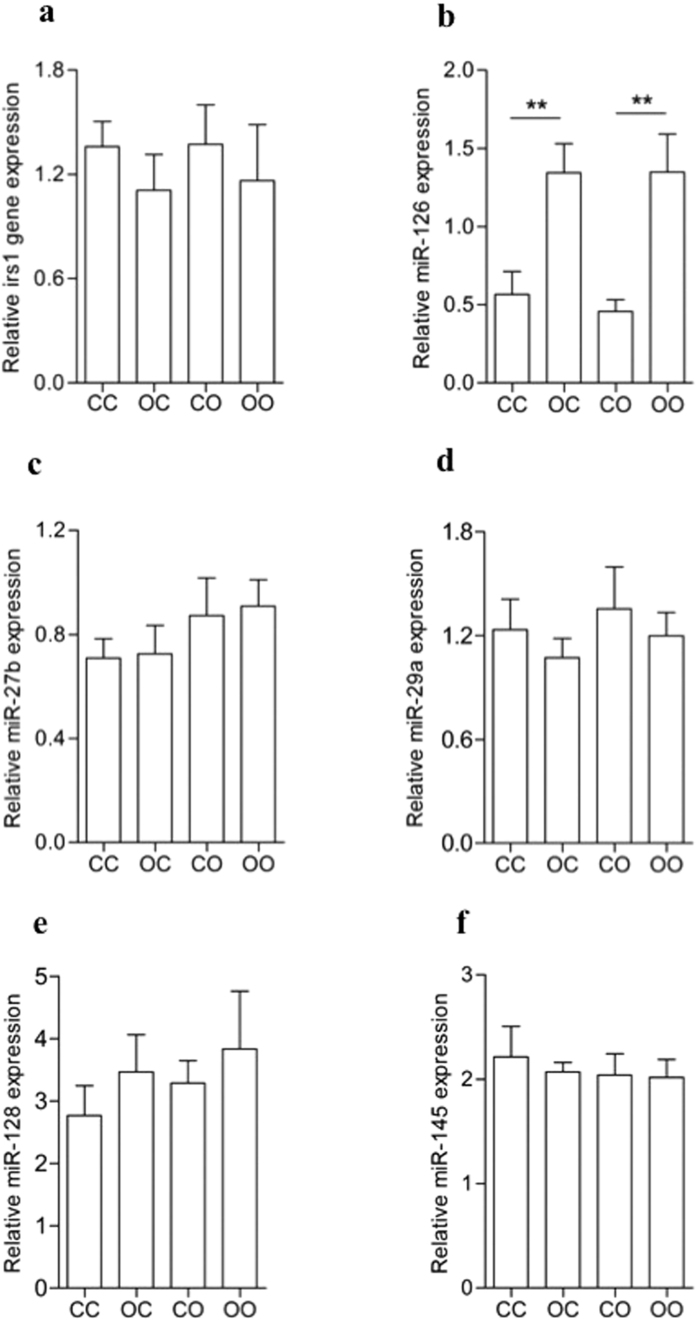
Maternal obesity but not post-weaning obesogenic diet regulates the miR-126 expression. (**a**) Irs1 mRNA levels were normalised by gapdh gene expression but no difference was found (n = 6). The miRNAs (**b**) miR-126, (**c**) miR-27b, (**d**) miR-29a, (**e**) miR-128 and (**f**) miR-145 were normalised by snRNA U6 expression (*n* = 6–8). Data are presented as mean ± SEM. Effect of maternal diet **p < 0.01.

Levels of *Irs1* mRNA were similar among all four groups (Fig. 5a) suggesting that the effects of both maternal and post-weaning diet-induced obesity operate through a post transcriptional mechanism(s) to decrease IRS-1 protein levels in offspring eWAT. As maternal obesity is already known to be associated with an increase in miR-126^14^ and IRS-1 is a validated target of this miRNA, the microRNA 126 was evaluated. Maternal obesity (p < 0.01) but not the post-weaning obesogenic diet altered miR-126 levels (Fig. 5b). Consistent with previous findings, miR-126 expression was significantly increased as a consequence of maternal obesity (p < 0.01) (Fig. 5b). However there was no effect of the offspring obesogenic diet on miR-126 levels. To establish if other miRNAs, which also have putative binding sites within the 3′UTR of IRS-1, could mediate the effects of the post weaning obesogenic diet on the offspring, we measured the expression of miRNA-27b (Fig. 5c), miR-29a (Fig. 5d), miR-128 (Fig. 5e) and miR-145 (Fig. 5f). However none of these miRNAs displayed any difference in expression compared to the control as a consequence of either the post-weaning or maternal obesogenic diet.

### Effects of maternal obesity and the post-weaning obesogenic diet on SOCS1 and SOCS3 expression

**Figure 6:**
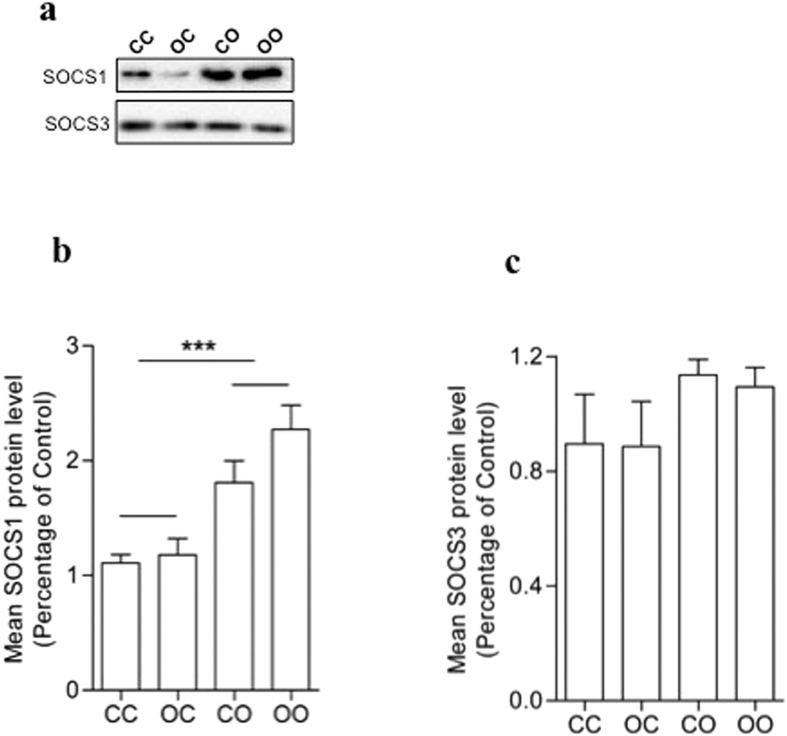
Obesogenic post-weaning diet but not maternal obesity raises SOCS1 expression in offspring eWAT. (**a**) Representative image of protein expression. (**b**) SOCS1 and (**c**) SOCS3 both represented as percentage of control group ± SEM (*n* = 4). Full-length blots are presented in Supplementary Figure 1b. Effect of offspring diet ***p < 0.001.

Previous studies have demonstrated that degradation pathways can play an important role in regulation of IRS-1 protein levels^19^. We therefore investigated the effects of the maternal and post-weaning obesogenic diet on the components of this degradation pathway, SOCS1 and SOCS3^20^,^21^. Offspring exposed to the post-weaning obesogenic diet (p < 0.001), but not offspring exposed to the maternal obesogenic diet, showed increased SOCS1 (Fig. 6b) protein levels in adipose tissue. SOCS3 (Fig. 6c) was not affected by either the maternal or post weaning obesogenic diet.

## Discussion

Obesity rates are increasing globally and this is associated with detrimental health consequences such as increased risk of type 2 diabetes and the metabolic syndrome. However some people appear to be more vulnerable to the effects of a current obesogenic environment than others and therefore more likely to experience these co-morbidities. In the present study, we hypothesised that exposure to maternal obesity whilst *in utero* would make an individual more susceptible to the detrimental effects of a postnatal obesogenic diet. We therefore investigated if weaning animals onto an obesogenic diet exacerbated the detrimental effects of maternal diet-induced obesity on offspring adipose tissue.

In young adult life (eight weeks of age) there was no effect of maternal diet-induced obesity on either body weight or epididymal fat pad weight of the offspring, consistent with our previous findings^14^. Weaning animals onto an obesogenic diet as expected led to an increase in body weight and fat pad weight which was accompanied by an increase in adipocyte cell size (hypertrophy) as is known to occur in situations of excessive energy consumption^22^. However, despite the lack of effect of maternal diet on fat pad weight, maternal obesity also led to an increase in adipocyte cell size. In addition both a maternal obesogenic diet and an offspring obesogenic diet led to an increase in lipid content of the adipose tissue. This suggests that both direct consumption of an obesogenic diet and maternal obesity *per se* can lead to remodeling of adipocytes. The effects of maternal obesity and current feeding of an obesogenic diet on adipocyte cell size were independent and additive thus animals exposed to both maternal obesity and a current obesogenic environment had the largest adipocytes. It has been shown previously that increased fat cell size is associated with insulin resistance^23^. Furthermore it has been demonstrated that non-obese individuals with type 2 diabetes have larger adipocytes in their subcutaneous white adipose tissue than glucose tolerant individuals^24^. These findings therefore suggest that individuals exposed to an obesogenic environment *in utero* are going to be at increased risk of developing type 2 diabetes when exposed to an obesogenic environment post natally.

Maternal obesity resulted in substantial reductions in a large number of insulin signaling proteins including the insulin receptor, IRS-1, the p110-beta catalytic subunit of PI 3-kinase, the p85alpha regulatory subunit of PI 3-kinase, AKT1 and AKT2. This was not a reflection of a global reduction in insulin signalling protein expression as PKCzeta and GLUT4 protein levels were unaffected by maternal obesity on the offspring at this age. Samuelsson *et al*. have shown that the development of glucose intolerance, as consequence of maternal obesity, occurs with age. In their study, offspring from obese dams at 3 months were insulin resistant and at 6 months they were hyperglycaemic with reduced fasting plasma insulin^17^. Furthermore, a previous study from our group has demonstrated that maternal diet-induced obesity leads to adipose tissue insulin resistance in the offspring before the development of obesity, which shows a clear effect of the maternal obesity on the offspring and the importance to investigate offspring at an early age (14). We have shown previously that IRS-1 and p110beta proteins are also reduced in adipose tissue from the offspring of low protein diet fed dams^25^ as well as low birthweight humans^26^. This suggests that programmed changes in these insulin signalling proteins occurs as a consequence of both maternal over-nutrition and maternal under-nutrition during pregnancy.

In the current study we demonstrated that a post weaning obesogenic diet also reduced expression of these same insulin signaling proteins. The effects of maternal obesity and a post-weaning obesogenic diet were additive, thus animals exposed to an obesogenic diet during both of these stages of life demonstrated the lowest expression levels of these insulin signalling proteins. The biggest combined effect was observed on IRS-1 protein that showed a 60% reduction in these animals compared to offspring of control dams that were weaned onto a control diet. Decreased IRS-1 expression in adipose tissue is known to reduce downstream insulin signaling through PI3K/AKT pathways causing a reduction in insulin-stimulated glucose uptake^27^. Therefore the combined effect of maternal obesity and a current obesogenic diet is likely to increase the risk of glucose intolerance. Interestingly, neither maternal obesity nor a post weaning obesogenic diet influenced *Irs1* mRNA levels. These findings suggest that the mechanisms underlying modulation of IRS-1 protein occur at a post-transcriptional level, which could involve changes in synthesis and/or degradation pathways.

miRNAs are small RNA species that through binding sequence specifically to regions in the 3′ un-translated region of mRNA act to reduce mRNA translation. Previously, we showed that maternal obesity results in increased miR-126 levels in offspring adipose tissue^14^. We^14^, and others^28^,^29^ have shown that miR-126 can directly regulate translation of IRS-1 mRNA. In the current study we confirmed up-regulation of miR-126 in response to maternal obesity. It is important to highlight that the effects of maternal diet on miR-126 and IRS-1 levels occur prior to any differences in adiposity. Therefore these changes are not a consequence of increased adiposity. It was for this reason that we chose the 8-week time point to study the animals to avoid confounding maternal effects on offspring obesity (that do not emerge in chow fed animals until later in life). Moreover we have shown at 8 weeks of age that offspring of obese dams when weaned onto chow are insulin resistant so this phenotype also develops independently of offspring obesity (14). In this study offspring were exposed to maternal obesity during fetal life and lactation, to reflect the fact that mothers who are obese during pregnancy are likely to still be obese during lactation. It is therefore not clear whether miR-126 levels are programmed during fetal or neonatal life or a combination of the two. Consumption of an obesogenic diet post-weaning had no effect on miR-126 levels or any other miRNAs known to regulate IRS-1 translation. *We therefore focused on degradation pathways as a potential mechanism to explain the effect of a post-weaning obesogenic diet on IRS-1 protein levels.* Previous studies in animals and humans have demonstrated that the rate of IRS-1 degradation in adipose tissue can be increased by obesity^30^ and/or type 2 diabetes^27^. Increased levels of SOCS proteins^19^,^20^ are thought to contribute to IRS-1 degradation. In the current study we demonstrated that an obesogenic diet post-weaning led to increased levels of SOCS1, a key component of the IRS-1 protein degradation pathway. There was no effect of maternal diet on SOCS1 levels. These findings suggest that maternal obesity and offspring obesity act through different pathways to reduce IRS-1 protein and therefore may explain why the effects are additive.

In summary, we have demonstrated for the first time that maternal diet-induced obesity followed by a post-natal obesogenic diet leads to a greater reduction in IRS-1 protein expression in offspring adipose tissue than maternal obesity alone. Furthermore, we demonstrate that reduction in IRS-1 occurs through two different post-transcriptional mechanisms depending on the timing of exposure to an obesogenic diet. Nutritional programing (as a consequence of maternal obesity) reduces IRS-1 translation through increased levels of mir126 whereas increased degradation of IRS-1 occurs in response to the postnatal obesogenic diet. Our findings also demonstrate that offspring from obese mothers have larger visceral adipocytes with higher fat content and impaired insulin signaling protein expression. These effects are exaggerated when these offspring are weaned onto an obesogenic diet. Consequently, individuals exposed to obesity *in utero* are more vulnerable to the detrimental effects of a post-weaning obesogenic diet and at greatest risk of developing type 2 diabetes. Such individuals are therefore an important group within the population to target intervention strategies. Our findings suggest that maternal obesity and a post-weaning obesogenic diet, at least in part, mediate their effects through different mechanisms and therefore provide more than one potential pathway for therapeutic intervention.

## Additional Information

**How to cite this article:** Faria, J. A. *et al*. A post-weaning obesogenic diet exacerbates the detrimental effects of maternal obesity on offspring insulin signaling in adipose tissue. *Sci. Rep.* **7**, 44949; doi: 10.1038/srep44949 (2017).

**Publisher's note:** Springer Nature remains neutral with regard to jurisdictional claims in published maps and institutional affiliations.

## Supplementary Material

Supplementary Information
