# Supplementary material for: A post-weaning obesogenic diet exacerbates the detrimental effects of maternal obesity on offspring insulin signaling in adipose tissue
Source: Sci Rep. 2017 Mar 24;7:44949. doi: 10.1038/srep44949 (PMC5364470; doi:10.1038/srep44949)
Supplement: Supplementary Information [file srep44949-s1.pdf]

Supplementary Information

Title: A post-weaning obesogenic diet exacerbates the detrimental effects of maternal obesity on offspring insulin signaling in adipose tissue.

Author and affiliations:

Juliana de Almeida Faria[1,2], Daniella E. Duque-Guimarães[1,3], Asha A. M. Carpenter[1], Elena Loche[1], Susan E. Ozanne[1]

[1] University of Cambridge Metabolic Research Laboratories and MRC Metabolic Diseases Unit, Wellcome Trust-MRC Institute of Metabolic Science, Addenbrooke's Hospital, Cambridge, CB2 0QQ, United Kingdom.

[2] University of Campinas, Faculty of Medical Sciences, Department of Pharmacology, Campinas, 13083-894, Brazil.

[3] University of São Paulo, Institute of Biomedical Sciences, Department of Physiology and Biophysics, São Paulo, 05508-000, Brazil.

a.

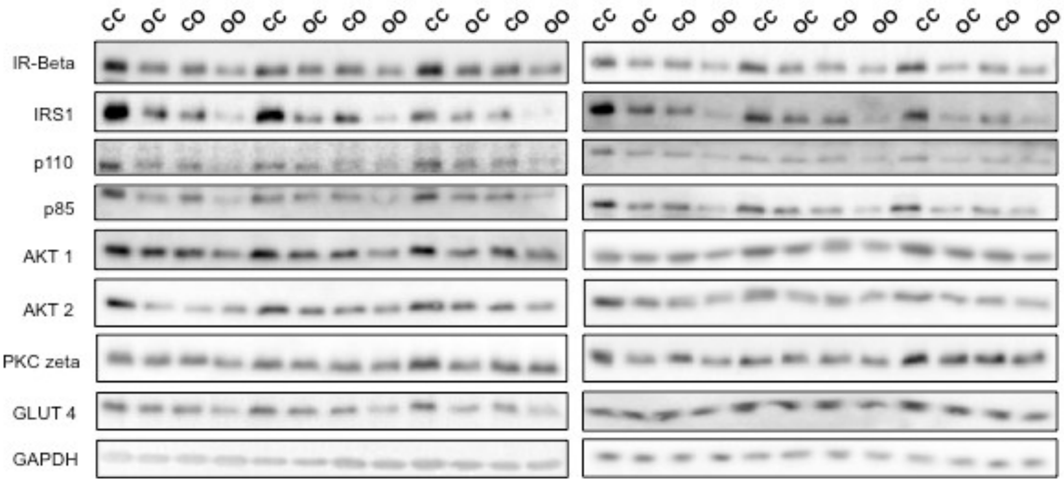

b.

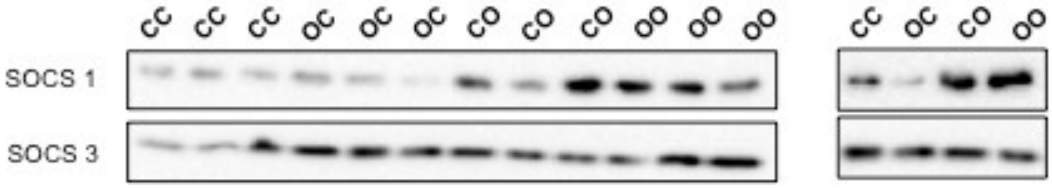

Figure S1: Full-length images of blots presented in the main figures.
